# Supplementary material for: Resolving the full spectrum of human genome variation using Linked-Reads
Source: Genome Res. 2019 Apr;29(4):635–45. doi: 10.1101/gr.234443.118 (PMC6442396; doi:10.1101/gr.234443.118)
Supplement: Supplemental Material [file supp_gr.234443.118_Supplemental_longranger-2.2.zip › longranger-2.2.tar.bz2!/lr2.2-public/lib/go/src/code.google.com/vitess/third_party/py/mock-1.0.1.tar.gz!/mock-1.0.1/html/changelog.html]

CHANGELOG — Mock 1.0.1 documentation


### Navigation

- index
- previous |
- Mock 1.0.1 documentation »

# CHANGELOG¶

## 2012/11/5 Version 1.0.1¶

- Functions decorated with patch variants have a \_\_wrapped\_\_ attribute
  pointing to the original function. This brings compatibility with the
  default behaviour in Python 3.3 (due to a new feature in functools.wraps).

Note that due to changes in tox, mock is no longer tested with Python 2.4.
The compatibility code has not been removed so it probably still works, but
tests are no longer run.

## 2012/10/07 Version 1.0.0¶

No changes since 1.0.0 beta 1. This version has feature parity with
unittest.mock
in Python 3.3.

Full list of changes since 0.8:

- mocksignature, along with the mocksignature argument to patch, removed
- Support for deleting attributes (accessing deleted attributes will raise an
  AttributeError)
- Added the mock\_open helper function for mocking the builtin open
- \_\_class\_\_ is assignable, so a mock can pass an isinstance check without
  requiring a spec
- Addition of PropertyMock, for mocking properties
- MagicMocks made unorderable by default (in Python 3). The comparison
  methods (other than equality and inequality) now return NotImplemented
- Propagate traceback info to support subclassing of \_patch by other
  libraries
- create\_autospec works with attributes present in results of dir that
  can’t be fetched from the object’s class. Contributed by Konstantine Rybnikov
- Any exceptions in an iterable side\_effect will be raised instead of
  returned
- In Python 3, create\_autospec now supports keyword only arguments
- Added patch.stopall method to stop all active patches created by start
- BUGFIX: calling MagicMock.reset\_mock wouldn’t reset magic method mocks
- BUGFIX: calling reset\_mock on a MagicMock created with autospec could
  raise an exception
- BUGFIX: passing multiple spec arguments to patchers (spec , spec\_set and
  autospec) had unpredictable results, now it is an error
- BUGFIX: using spec=True *and* create=True as arguments to patchers could
  result in using DEFAULT as the spec. Now it is an error instead
- BUGFIX: using spec or autospec arguments to patchers, along with
  spec\_set=True did not work correctly
- BUGFIX: using an object that evaluates to False as a spec could be ignored
- BUGFIX: a list as the spec argument to a patcher would always result in a
  non-callable mock. Now if \_\_call\_\_ is in the spec the mock is callable

## 2012/07/13 Version 1.0.0 beta 1¶

- Added patch.stopall method to stop all active patches created by start
- BUGFIX: calling MagicMock.reset\_mock wouldn’t reset magic method mocks
- BUGFIX: calling reset\_mock on a MagicMock created with autospec could
  raise an exception

## 2012/05/04 Version 1.0.0 alpha 2¶

- PropertyMock attributes are now standard MagicMocks
- create\_autospec works with attributes present in results of dir that
  can’t be fetched from the object’s class. Contributed by Konstantine Rybnikov
- Any exceptions in an iterable side\_effect will be raised instead of
  returned
- In Python 3, create\_autospec now supports keyword only arguments

## 2012/03/25 Version 1.0.0 alpha 1¶

The standard library version!

- mocksignature, along with the mocksignature argument to patch, removed
- Support for deleting attributes (accessing deleted attributes will raise an
  AttributeError)
- Added the mock\_open helper function for mocking the builtin open
- \_\_class\_\_ is assignable, so a mock can pass an isinstance check without
  requiring a spec
- Addition of PropertyMock, for mocking properties
- MagicMocks made unorderable by default (in Python 3). The comparison
  methods (other than equality and inequality) now return NotImplemented
- Propagate traceback info to support subclassing of \_patch by other
  libraries
- BUGFIX: passing multiple spec arguments to patchers (spec , spec\_set and
  autospec) had unpredictable results, now it is an error
- BUGFIX: using spec=True *and* create=True as arguments to patchers could
  result in using DEFAULT as the spec. Now it is an error instead
- BUGFIX: using spec or autospec arguments to patchers, along with
  spec\_set=True did not work correctly
- BUGFIX: using an object that evaluates to False as a spec could be ignored
- BUGFIX: a list as the spec argument to a patcher would always result in a
  non-callable mock. Now if \_\_call\_\_ is in the spec the mock is callable

## 2012/02/13 Version 0.8.0¶

The only changes since 0.8rc2 are:

- Improved repr of sentinel objects
- ANY can be used for comparisons against call objects
- The return value of MagicMock.\_\_iter\_\_ method can be set to
  any iterable and isn’t required to be an iterator

Full List of changes since 0.7:

mock 0.8.0 is the last version that will support Python 2.4.

- Addition of mock\_calls list for *all* calls (including magic
  methods and chained calls)
- patch() and patch.object() now create a MagicMock
  instead of a Mock by default
- The patchers (patch, patch.object and patch.dict), plus Mock and
  MagicMock, take arbitrary keyword arguments for configuration
- New mock method configure\_mock() for setting attributes and
  return values / side effects on the mock and its attributes
- New mock assert methods assert\_any\_call() and
  assert\_has\_calls()
- Implemented *Autospeccing* (recursive, lazy speccing of mocks with
  mocked signatures for functions/methods), as the autospec argument to
  patch
- Added the create\_autospec() function for manually creating
  ‘auto-specced’ mocks
- patch.multiple() for doing multiple patches in a single call, using
  keyword arguments
- Setting side\_effect to an iterable will cause calls to the mock
  to return the next value from the iterable
- New new\_callable argument to patch and patch.object allowing you to
  pass in a class or callable object (instead of MagicMock) that will be
  called to replace the object being patched
- Addition of NonCallableMock and NonCallableMagicMock, mocks
  without a \_\_call\_\_ method
- Addition of mock\_add\_spec() method for adding (or changing) a
  spec on an existing mock
- Protocol methods on MagicMock are magic mocks, and are created
  lazily on first lookup. This means the result of calling a protocol method is
  a MagicMock instead of a Mock as it was previously
- Addition of attach\_mock() method
- Added ANY for ignoring arguments in assert\_called\_with()
  calls
- Addition of call helper object
- Improved repr for mocks
- Improved repr for Mock.call\_args and entries in
  Mock.call\_args\_list, Mock.method\_calls and
  Mock.mock\_calls
- Improved repr for sentinel objects
- patch lookup is done at use time not at decoration time
- In Python 2.6 or more recent, dir on a mock will report all the dynamically
  created attributes (or the full list of attributes if there is a spec) as
  well as all the mock methods and attributes.
- Module level FILTER\_DIR added to control whether dir(mock) filters
  private attributes. True by default.
- patch.TEST\_PREFIX for controlling how patchers recognise test methods when
  used to decorate a class
- Support for using Java exceptions as a side\_effect on Jython
- Mock call lists (call\_args\_list, method\_calls & mock\_calls) are now
  custom list objects that allow membership tests for “sub lists” and have
  a nicer representation if you str or print them
- Mocks attached as attributes or return values to other mocks have calls
  recorded in method\_calls and mock\_calls of the parent (unless a name is
  already set on the child)
- Improved failure messages for assert\_called\_with and
  assert\_called\_once\_with
- The return value of the MagicMock \_\_iter\_\_ method can be set to
  any iterable and isn’t required to be an iterator
- Added the Mock API (assert\_called\_with etc) to functions created by
  mocksignature()
- Tuples as well as lists can be used to specify allowed methods for spec &
  spec\_set arguments
- Calling stop on an unstarted patcher fails with a more meaningful error
  message
- Renamed the internal classes Sentinel and SentinelObject to prevent abuse
- BUGFIX: an error creating a patch, with nested patch decorators, won’t leave
  patches in place
- BUGFIX: \_\_truediv\_\_ and \_\_rtruediv\_\_ not available as magic methods on
  mocks in Python 3
- BUGFIX: assert\_called\_with / assert\_called\_once\_with can be used with
  self as a keyword argument
- BUGFIX: when patching a class with an explicit spec / spec\_set (not a
  boolean) it applies “spec inheritance” to the return value of the created
  mock (the “instance”)
- BUGFIX: remove the \_\_unittest marker causing traceback truncation
- Removal of deprecated patch\_object
- Private attributes \_name, \_methods, ‘\_children’, \_wraps and \_parent
  (etc) renamed to reduce likelihood of clash with user attributes.
- Added license file to the distribution

## 2012/01/10 Version 0.8.0 release candidate 2¶

- Removed the configure keyword argument to create\_autospec and allow
  arbitrary keyword arguments (for the Mock constructor) instead
- Fixed ANY equality with some types in assert\_called\_with calls
- Switched to a standard Sphinx theme (compatible with
  readthedocs.org)

## 2011/12/29 Version 0.8.0 release candidate 1¶

- create\_autospec on the return value of a mocked class will use \_\_call\_\_
  for the signature rather than \_\_init\_\_
- Performance improvement instantiating Mock and MagicMock
- Mocks used as magic methods have the same type as their parent instead of
  being hardcoded to MagicMock

Special thanks to Julian Berman for his help with diagnosing and improving
performance in this release.

## 2011/10/09 Version 0.8.0 beta 4¶

- patch lookup is done at use time not at decoration time
- When attaching a Mock to another Mock as a magic method, calls are recorded
  in mock\_calls
- Addition of attach\_mock method
- Renamed the internal classes Sentinel and SentinelObject to prevent abuse
- BUGFIX: various issues around circular references with mocks (setting a mock
  return value to be itself etc)

## 2011/08/15 Version 0.8.0 beta 3¶

- Mocks attached as attributes or return values to other mocks have calls
  recorded in method\_calls and mock\_calls of the parent (unless a name is
  already set on the child)
- Addition of mock\_add\_spec method for adding (or changing) a spec on an
  existing mock
- Improved repr for Mock.call\_args and entries in Mock.call\_args\_list,
  Mock.method\_calls and Mock.mock\_calls
- Improved repr for mocks
- BUGFIX: minor fixes in the way mock\_calls is worked out,
  especially for “intermediate” mocks in a call chain

## 2011/08/05 Version 0.8.0 beta 2¶

- Setting side\_effect to an iterable will cause calls to the mock to return
  the next value from the iterable
- Added assert\_any\_call method
- Moved assert\_has\_calls from call lists onto mocks
- BUGFIX: call\_args and all members of call\_args\_list are two tuples of
  (args, kwargs) again instead of three tuples of (name, args, kwargs)

## 2011/07/25 Version 0.8.0 beta 1¶

- patch.TEST\_PREFIX for controlling how patchers recognise test methods when
  used to decorate a class
- Mock call lists (call\_args\_list, method\_calls & mock\_calls) are now
  custom list objects that allow membership tests for “sub lists” and have
  an assert\_has\_calls method for unordered call checks
- callargs changed to *always* be a three-tuple of (name, args, kwargs)
- Addition of mock\_calls list for *all* calls (including magic methods and
  chained calls)
- Extension of call object to support chained calls and callargs for better
  comparisons with or without names. call object has a call\_list method for
  chained calls
- Added the public instance argument to create\_autospec
- Support for using Java exceptions as a side\_effect on Jython
- Improved failure messages for assert\_called\_with and
  assert\_called\_once\_with
- Tuples as well as lists can be used to specify allowed methods for spec &
  spec\_set arguments
- BUGFIX: Fixed bug in patch.multiple for argument passing when creating
  mocks
- Added license file to the distribution

## 2011/07/16 Version 0.8.0 alpha 2¶

- patch.multiple for doing multiple patches in a single call, using keyword
  arguments
- New new\_callable argument to patch and patch.object allowing you to
  pass in a class or callable object (instead of MagicMock) that will be
  called to replace the object being patched
- Addition of NonCallableMock and NonCallableMagicMock, mocks without a
  \_\_call\_\_ method
- Mocks created by patch have a MagicMock as the return\_value where a
  class is being patched
- create\_autospec can create non-callable mocks for non-callable objects.
  return\_value mocks of classes will be non-callable unless the class has
  a \_\_call\_\_ method
- autospec creates a MagicMock without a spec for properties and slot
  descriptors, because we don’t know the type of object they return
- Removed the “inherit” argument from create\_autospec
- Calling stop on an unstarted patcher fails with a more meaningful error
  message
- BUGFIX: an error creating a patch, with nested patch decorators, won’t leave
  patches in place
- BUGFIX: \_\_truediv\_\_ and \_\_rtruediv\_\_ not available as magic methods on
  mocks in Python 3
- BUGFIX: assert\_called\_with / assert\_called\_once\_with can be used with
  self as a keyword argument
- BUGFIX: autospec for functions / methods with an argument named self that
  isn’t the first argument no longer broken
- BUGFIX: when patching a class with an explicit spec / spec\_set (not a
  boolean) it applies “spec inheritance” to the return value of the created
  mock (the “instance”)
- BUGFIX: remove the \_\_unittest marker causing traceback truncation

## 2011/06/14 Version 0.8.0 alpha 1¶

mock 0.8.0 is the last version that will support Python 2.4.

- The patchers (patch, patch.object and patch.dict), plus Mock and
  MagicMock, take arbitrary keyword arguments for configuration
- New mock method configure\_mock for setting attributes and return values /
  side effects on the mock and its attributes
- In Python 2.6 or more recent, dir on a mock will report all the dynamically
  created attributes (or the full list of attributes if there is a spec) as
  well as all the mock methods and attributes.
- Module level FILTER\_DIR added to control whether dir(mock) filters
  private attributes. True by default. Note that vars(Mock()) can still be
  used to get all instance attributes and dir(type(Mock()) will still return
  all the other attributes (irrespective of FILTER\_DIR)
- patch and patch.object now create a MagicMock instead of a Mock by
  default
- Added ANY for ignoring arguments in assert\_called\_with calls
- Addition of call helper object
- Protocol methods on MagicMock are magic mocks, and are created lazily on
  first lookup. This means the result of calling a protocol method is a
  MagicMock instead of a Mock as it was previously
- Added the Mock API (assert\_called\_with etc) to functions created by
  mocksignature
- Private attributes \_name, \_methods, ‘\_children’, \_wraps and \_parent
  (etc) renamed to reduce likelihood of clash with user attributes.
- Implemented auto-speccing (recursive, lazy speccing of mocks with mocked
  signatures for functions/methods)

  Limitations:

  - Doesn’t mock magic methods or attributes (it creates MagicMocks, so the
    magic methods are *there*, they just don’t have the signature mocked nor
    are attributes followed)
  - Doesn’t mock function / method attributes
  - Uses object traversal on the objects being mocked to determine types - so
    properties etc may be triggered
  - The return value of mocked classes (the ‘instance’) has the same call
    signature as the class \_\_init\_\_ (as they share the same spec)

  You create auto-specced mocks by passing autospec=True to patch.

  Note that attributes that are None are special cased and mocked without a
  spec (so any attribute / method can be used). This is because None is
  typically used as a default value for attributes that may be of some other
  type, and as we don’t know what type that may be we allow all access.

  Note that the autospec option to patch obsoletes the mocksignature
  option.
- Added the create\_autospec function for manually creating ‘auto-specced’
  mocks
- Removal of deprecated patch\_object

## 2011/05/30 Version 0.7.2¶

- BUGFIX: instances of list subclasses can now be used as mock specs
- BUGFIX: MagicMock equality / inequality protocol methods changed to use the
  default equality / inequality. This is done through a side\_effect on
  the mocks used for \_\_eq\_\_ / \_\_ne\_\_

## 2011/05/06 Version 0.7.1¶

Package fixes contributed by Michael Fladischer. No code changes.

- Include template in package
- Use isolated binaries for the tox tests
- Unset executable bit on docs
- Fix DOS line endings in getting-started.txt

## 2011/03/05 Version 0.7.0¶

No API changes since 0.7.0 rc1. Many documentation changes including a stylish
new Sphinx theme.

The full set of changes since 0.6.0 are:

- Python 3 compatibility
- Ability to mock magic methods with Mock and addition of MagicMock
  with pre-created magic methods
- Addition of mocksignature and mocksignature argument to patch and
  patch.object
- Addition of patch.dict for changing dictionaries during a test
- Ability to use patch, patch.object and patch.dict as class decorators
- Renamed patch\_object to patch.object (patch\_object is
  deprecated)
- Addition of soft comparisons: call\_args, call\_args\_list and method\_calls
  now return tuple-like objects which compare equal even when empty args
  or kwargs are skipped
- patchers (patch, patch.object and patch.dict) have start and stop
  methods
- Addition of assert\_called\_once\_with method
- Mocks can now be named (name argument to constructor) and the name is used
  in the repr
- repr of a mock with a spec includes the class name of the spec
- assert\_called\_with works with python -OO
- New spec\_set keyword argument to Mock and patch. If used,
  attempting to *set* an attribute on a mock not on the spec will raise an
  AttributeError
- Mocks created with a spec can now pass isinstance tests (\_\_class\_\_
  returns the type of the spec)
- Added docstrings to all objects
- Improved failure message for Mock.assert\_called\_with when the mock
  has not been called at all
- Decorated functions / methods have their docstring and \_\_module\_\_
  preserved on Python 2.4.
- BUGFIX: mock.patch now works correctly with certain types of objects that
  proxy attribute access, like the django settings object
- BUGFIX: mocks are now copyable (thanks to Ned Batchelder for reporting and
  diagnosing this)
- BUGFIX: spec=True works with old style classes
- BUGFIX: help(mock) works now (on the module). Can no longer use \_\_bases\_\_
  as a valid sentinel name (thanks to Stephen Emslie for reporting and
  diagnosing this)
- BUGFIX: side\_effect now works with BaseException exceptions like
  KeyboardInterrupt
- BUGFIX: reset\_mock caused infinite recursion when a mock is set as its own
  return value
- BUGFIX: patching the same object twice now restores the patches correctly
- with statement tests now skipped on Python 2.4
- Tests require unittest2 (or unittest2-py3k) to run
- Tested with tox on Python 2.4 - 3.2,
  jython and pypy (excluding 3.0)
- Added ‘build\_sphinx’ command to setup.py (requires setuptools or distribute)
  Thanks to Florian Bauer
- Switched from subversion to mercurial for source code control
- Konrad Delong added as co-maintainer

## 2011/02/16 Version 0.7.0 RC 1¶

Changes since beta 4:

- Tested with jython, pypy and Python 3.2 and 3.1
- Decorated functions / methods have their docstring and \_\_module\_\_
  preserved on Python 2.4
- BUGFIX: mock.patch now works correctly with certain types of objects that
  proxy attribute access, like the django settings object
- BUGFIX: reset\_mock caused infinite recursion when a mock is set as its own
  return value

## 2010/11/12 Version 0.7.0 beta 4¶

- patchers (patch, patch.object and patch.dict) have start and stop
  methods
- Addition of assert\_called\_once\_with method
- repr of a mock with a spec includes the class name of the spec
- assert\_called\_with works with python -OO
- New spec\_set keyword argument to Mock and patch. If used,
  attempting to *set* an attribute on a mock not on the spec will raise an
  AttributeError
- Attributes and return value of a MagicMock are MagicMock objects
- Attempting to set an unsupported magic method now raises an AttributeError
- patch.dict works as a class decorator
- Switched from subversion to mercurial for source code control
- BUGFIX: mocks are now copyable (thanks to Ned Batchelder for reporting and
  diagnosing this)
- BUGFIX: spec=True works with old style classes
- BUGFIX: mocksignature=True can now patch instance methods via
  patch.object

## 2010/09/18 Version 0.7.0 beta 3¶

- Using spec with MagicMock only pre-creates magic methods in the spec
- Setting a magic method on a mock with a spec can only be done if the
  spec has that method
- Mocks can now be named (name argument to constructor) and the name is used
  in the repr
- mocksignature can now be used with classes (signature based on \_\_init\_\_)
  and callable objects (signature based on \_\_call\_\_)
- Mocks created with a spec can now pass isinstance tests (\_\_class\_\_
  returns the type of the spec)
- Default numeric value for MagicMock is 1 rather than zero (because the
  MagicMock bool defaults to True and 0 is False)
- Improved failure message for assert\_called\_with() when the mock
  has not been called at all
- Adding the following to the set of supported magic methods:
  - \_\_getformat\_\_ and \_\_setformat\_\_
  - pickle methods
  - \_\_trunc\_\_, \_\_ceil\_\_ and \_\_floor\_\_
  - \_\_sizeof\_\_
- Added ‘build\_sphinx’ command to setup.py (requires setuptools or distribute)
  Thanks to Florian Bauer
- with statement tests now skipped on Python 2.4
- Tests require unittest2 to run on Python 2.7
- Improved several docstrings and documentation

## 2010/06/23 Version 0.7.0 beta 2¶

- patch.dict() works as a context manager as well as a decorator
- patch.dict takes a string to specify dictionary as well as a dictionary
  object. If a string is supplied the name specified is imported
- BUGFIX: patch.dict restores dictionary even when an exception is raised

## 2010/06/22 Version 0.7.0 beta 1¶

- Addition of mocksignature()
- Ability to mock magic methods
- Ability to use patch and patch.object as class decorators
- Renamed patch\_object to patch.object() (patch\_object is
  deprecated)
- Addition of MagicMock class with all magic methods pre-created for you
- Python 3 compatibility (tested with 3.2 but should work with 3.0 & 3.1 as
  well)
- Addition of patch.dict() for changing dictionaries during a test
- Addition of mocksignature argument to patch and patch.object
- help(mock) works now (on the module). Can no longer use \_\_bases\_\_
  as a valid sentinel name (thanks to Stephen Emslie for reporting and
  diagnosing this)
- Addition of soft comparisons: call\_args, call\_args\_list and method\_calls
  now return tuple-like objects which compare equal even when empty args
  or kwargs are skipped
- Added docstrings.
- BUGFIX: side\_effect now works with BaseException exceptions like
  KeyboardInterrupt
- BUGFIX: patching the same object twice now restores the patches correctly
- The tests now require unittest2
  to run
- Konrad Delong added as co-maintainer

## 2009/08/22 Version 0.6.0¶

- New test layout compatible with test discovery
- Descriptors (static methods / class methods etc) can now be patched and
  restored correctly
- Mocks can raise exceptions when called by setting side\_effect to an
  exception class or instance
- Mocks that wrap objects will not pass on calls to the underlying object if
  an explicit return\_value is set

## 2009/04/17 Version 0.5.0¶

- Made DEFAULT part of the public api.
- Documentation built with Sphinx.
- side\_effect is now called with the same arguments as the mock is called with and
  if returns a non-DEFAULT value that is automatically set as the mock.return\_value.
- wraps keyword argument used for wrapping objects (and passing calls through to the wrapped object).
- Mock.reset renamed to Mock.reset\_mock, as reset is a common API name.
- patch / patch\_object are now context managers and can be used with with.
- A new ‘create’ keyword argument to patch and patch\_object that allows them to patch
  (and unpatch) attributes that don’t exist. (Potentially unsafe to use - it can allow
  you to have tests that pass when they are testing an API that doesn’t exist - use at
  your own risk!)
- The methods keyword argument to Mock has been removed and merged with spec. The spec
  argument can now be a list of methods or an object to take the spec from.
- Nested patches may now be applied in a different order (created mocks passed
  in the opposite order). This is actually a bugfix.
- patch and patch\_object now take a spec keyword argument. If spec is
  passed in as ‘True’ then the Mock created will take the object it is replacing
  as its spec object. If the object being replaced is a class, then the return
  value for the mock will also use the class as a spec.
- A Mock created without a spec will not attempt to mock any magic methods / attributes
  (they will raise an AttributeError instead).

## 2008/10/12 Version 0.4.0¶

- Default return value is now a new mock rather than None
- return\_value added as a keyword argument to the constructor
- New method ‘assert\_called\_with’
- Added ‘side\_effect’ attribute / keyword argument called when mock is called
- patch decorator split into two decorators:

  > - patch\_object which takes an object and an attribute name to patch
  >   (plus optionally a value to patch with which defaults to a mock object)
  > - patch which takes a string specifying a target to patch; in the form
  >   ‘package.module.Class.attribute’. (plus optionally a value to
  >   patch with which defaults to a mock object)
- Can now patch objects with None
- Change to patch for nose compatibility with error reporting in wrapped functions
- Reset no longer clears children / return value etc - it just resets
  call count and call args. It also calls reset on all children (and
  the return value if it is a mock).

Thanks to Konrad Delong, Kevin Dangoor and others for patches and suggestions.

## 2007/12/03 Version 0.3.1¶

patch maintains the name of decorated functions for compatibility with nose
test autodiscovery.

Tests decorated with patch that use the two argument form (implicit mock
creation) will receive the mock(s) passed in as extra arguments.

Thanks to Kevin Dangoor for these changes.

## 2007/11/30 Version 0.3.0¶

Removed patch\_module. patch can now take a string as the first
argument for patching modules.

The third argument to patch is optional - a mock will be created by
default if it is not passed in.

## 2007/11/21 Version 0.2.1¶

Bug fix, allows reuse of functions decorated with patch and patch\_module.

## 2007/11/20 Version 0.2.0¶

Added spec keyword argument for creating Mock objects from a
specification object.

Added patch and patch\_module monkey patching decorators.

Added sentinel for convenient access to unique objects.

Distribution includes unit tests.

## 2007/11/19 Version 0.1.0¶

Initial release.

# TODO and Limitations¶

Contributions, bug reports and comments welcomed!

Feature requests and bug reports are handled on the issue tracker:

> - mock issue tracker

wraps is not integrated with magic methods.

patch could auto-do the patching in the constructor and unpatch in the
destructor. This would be useful in itself, but violates TOOWTDI and would be
unsafe for IronPython & PyPy (non-deterministic calling of destructors).
Destructors aren’t called in CPython where there are cycles, but a weak
reference with a callback can be used to get round this.

Mock has several attributes. This makes it unsuitable for mocking objects
that use these attribute names. A way round this would be to provide methods
that *hide* these attributes when needed. In 0.8 many, but not all, of these
attributes are renamed to gain a \_mock prefix, making it less likely that
they will clash. Any outstanding attributes that haven’t been modified with
the prefix should be changed.

If a patch is started using patch.start and then not stopped correctly then
the unpatching is not done. Using weak references it would be possible to
detect and fix this when the patch object itself is garbage collected. This
would be tricky to get right though.

When a Mock is created by patch, arbitrary keywords can be used to set
attributes. If patch is created with a spec, and is replacing a class, then
a return\_value mock is created. The keyword arguments are not applied to the
child mock, but could be.

When mocking a class with patch, passing in spec=True or autospec=True,
the mock class has an instance created from the same spec. Should this be the
default behaviour for mocks anyway (mock return values inheriting the spec
from their parent), or should it be controlled by an additional keyword
argument (inherit) to the Mock constructor? create\_autospec does this, so
an additional keyword argument to Mock is probably unnecessary.

The mocksignature argument to patch with a non Mock passed into
new\_callable will *probably* cause an error. Should it just be invalid?

Note that NonCallableMock and NonCallableMagicMock still have the unused
(and unusable) attributes: return\_value, side\_effect, call\_count,
call\_args and call\_args\_list. These could be removed or raise errors on
getting / setting. They also have the assert\_called\_with and
assert\_called\_once\_with methods. Removing these would be pointless as
fetching them would create a mock (attribute) that could be called without
error.

Some outstanding technical debt. The way autospeccing mocks function
signatures was copied and modified from mocksignature. This could all be
refactored into one set of functions instead of two. The way we tell if
patchers are started and if a patcher is being used for a patch.multiple
call are both horrible. There are now a host of helper functions that should
be rationalised. (Probably time to split mock into a package instead of a
module.)

Passing arbitrary keyword arguments to create\_autospec, or patch with
autospec, when mocking a *function* works fine. However, the arbitrary
attributes are set on the created mock - but create\_autospec returns a
real function (which doesn’t have those attributes). However, what is the use
case for using autospec to create functions with attributes that don’t exist
on the original?

mocksignature, plus the call\_args\_list and method\_calls attributes of
Mock could all be deprecated.

### Table Of Contents

- CHANGELOG
  - 2012/11/5 Version 1.0.1
  - 2012/10/07 Version 1.0.0
  - 2012/07/13 Version 1.0.0 beta 1
  - 2012/05/04 Version 1.0.0 alpha 2
  - 2012/03/25 Version 1.0.0 alpha 1
  - 2012/02/13 Version 0.8.0
  - 2012/01/10 Version 0.8.0 release candidate 2
  - 2011/12/29 Version 0.8.0 release candidate 1
  - 2011/10/09 Version 0.8.0 beta 4
  - 2011/08/15 Version 0.8.0 beta 3
  - 2011/08/05 Version 0.8.0 beta 2
  - 2011/07/25 Version 0.8.0 beta 1
  - 2011/07/16 Version 0.8.0 alpha 2
  - 2011/06/14 Version 0.8.0 alpha 1
  - 2011/05/30 Version 0.7.2
  - 2011/05/06 Version 0.7.1
  - 2011/03/05 Version 0.7.0
  - 2011/02/16 Version 0.7.0 RC 1
  - 2010/11/12 Version 0.7.0 beta 4
  - 2010/09/18 Version 0.7.0 beta 3
  - 2010/06/23 Version 0.7.0 beta 2
  - 2010/06/22 Version 0.7.0 beta 1
  - 2009/08/22 Version 0.6.0
  - 2009/04/17 Version 0.5.0
  - 2008/10/12 Version 0.4.0
  - 2007/12/03 Version 0.3.1
  - 2007/11/30 Version 0.3.0
  - 2007/11/21 Version 0.2.1
  - 2007/11/20 Version 0.2.0
  - 2007/11/19 Version 0.1.0
- TODO and Limitations

#### Previous topic

Mock Library Comparison

### This Page

- Show Source

### Quick search


Enter search terms or a module, class or function name.

### Navigation

- index
- previous |
- Mock 1.0.1 documentation »

© Copyright 2007-2012, Michael Foord & the mock team.
Last updated on Nov 05, 2012.
Created using Sphinx 1.1.3.
